# Primary Multifocal Recurrent Urinary Bladder Amyloidosis: A Rare Case Report from Syria

**DOI:** 10.1155/2021/4476363

**Published:** 2021-10-22

**Authors:** Leen Jamel Doya, Lama Doya, Khaled Al-Yousef, Ali Adib, Abedallah Al Shamali, Basel Al Deeb, Mohamad Kanan

**Affiliations:** ^1^Department of Pediatrics, Tishreen University Hospital, Lattakia, Syria; ^2^Department of Laboratory, Tishreen University Hospital, Faculty of Medicine, Lattakia, Syria; ^3^Department of Urology, Tishreen University Hospital, Lattakia, Syria

## Abstract

Primary urinary bladder amyloidosis is a rare disorder of protein metabolism characterized by the extracellular deposition of fibrillin. To date, fewer than 200 cases have been reported in the literature. We herein present a case of 59-year-old female with primary multifocal recurrent urinary bladder amyloidosis. The patient was treated with a new method (laser therapy) mentioned for the first time in the literature. After 18 months of treatment, the patient has no complaints. Our case illustrates a new procedure in the treatment of primary multifocal bladder amyloidosis.

## 1. Introduction

Amyloidosis is a heterogeneous group disorder characterized by the extracellular deposition of eosinophilic fibrillar protein (amyloid protein) in various tissues and organs [1]. Historically, the term amyloid was coined for the first time by Virchow in 1854. In 1897, Solomin described the first clinical case of bladder amyloidosis (BA) at autopsy [2]. BA is most frequent in the 5^th^ and 6^th^ decade with equal gender preponderance [3].

There are two forms of BA: primary urinary bladder amyloidosis (PUBA) and secondary (commonly associated with chronic inflammatory conditions) [1]. Gross hematuria with irritative lower urinary tract symptoms is the most prominent in PUBA. It may be easily confused with bladder cancer [3]. There are no standard management protocols of PUBA due to its rarity. Herein we present the first rare case in Syria of recurrent multifocal PUBA treated with laser therapy for the first time in the medical literature.

## 2. Case Report

A 59-year-old Syrian female was admitted with a 2-month history of gross hematuria associated with lower urinary tract symptoms. There were no systemic symptoms (fever, joint pain, or rash). The hematuria was red in color, fresh, and intermittent with no clotting. She was diagnosed with PUBA 18 years ago treated by transurethral resection without follow-up.

The physical examinations were unremarkable. Complete blood counts (CBCs) and routine biochemical tests (renal, liver, and bone parameters) were normal. Urinary microscopic examination showed normal pH and specific gravity, with uncountable RBCs, few WBCs, granules casts, and presence of hemoglobin. Culture and sensitivity were normal.

**Figure 1:**
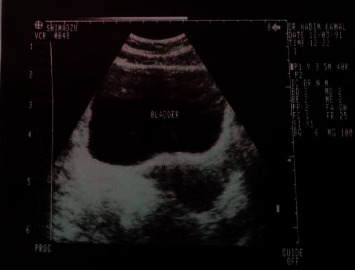
Ultrasound scan of the kidneys, ureters, and bladder showed thickening and irregularity of the bladder wall.

**Figure 2:**
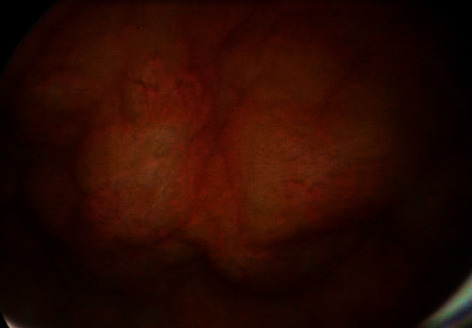
Cystoscopy showed multifocal yellowish bladder lesions.

Ultrasound of the kidneys, ureters, and bladder showed thickening and irregularity of the bladder wall with 9 mm as the maximum thickness (Figure 1). Intravenous urography was normal. Cystoscopy showed multifocal yellowish bladder lesions (Figure 2). The pathologic examination demonstrated extensive deposits of a cellular amorphous compatible with amyloidosis. No other abnormality was detected.

To exclude systemic amyloidosis, an electrocardiogram (ECG) and chest X-ray were conducted which revealed no pathologic findings. Tuberculin reaction and tuberculosis screening in 24 hours urine were normal. There was no increased lymphocyte count and protein gap. Bone marrow examination and CT scan of abdomen were both normal.

After the exclusion of systemic amyloidosis, recurrent multifocal PUBA diagnosis was confirmed.

A new method in the treatment of PUBA with neodymium: yttrium-aluminum garnet (Nd: YAG) laser therapy had been performed. The laser intensity used was 25 Watt with pulse duration less than 3 seconds. The fibreoptic tip was placed 1–3 mm from the bladder wall using a spot size of 2 mm. The total energy was 23500 Joules.

After 18 months from treatment, the patient was normal without symptoms. She has since remained asymptomatic without progression of systemic amyloidosis. Follow-up cystoscopies performed at present have been normal.

## 3. Discussion

The underlying mechanism of the PUBA is still controversial. Chronic mucosal and submucosal inflammation results in infiltration of lymphoplasmacytic cells with subsequent amyloid fibril deposition [4].

Computed tomography (CT) and magnetic resonance imaging (MRI) are not always useful in differentiating amyloidosis from other pathological conditions. CT shows thickening of the bladder wall, a mass lesion, or a filling defect in the urinary bladder which leads to the differential diagnosis of invasive bladder tumor or inflammatory lesion [1]. Cystoscopy showed nodular or polypoidal lesions that mimic the appearance of primary urothelial carcinomas. The gold standard of the PUBA diagnosis is bladder biopsy [5].

To date, approximately 210 cases of BUPA have been reported in the literature (over 22 countries; 60 cases in the UK). The current case report is the first report from Syria that described a case of recurrent multifocal PUBA treated with laser therapy.

In the literature, transurethral resection or partial cystectomy is the most used option for treating PUBA with a high rate of complication and recurrence [6]. None of these cases used laser therapy as the first choice of treatment for the recurrent multifocal cases. Despite the wide multifocal lesions of the PUBA in the current case, laser was used for the first time over wide lesions without any significant complaint.

There are rare reports of conservative treatment such as intravesical dimethyl sulfoxide installation and oral medications (colchicine) in the smaller lesions. Laser therapy or fulguration has been used for small, localized lesions [4]. Long-term cystoscopic surveillance with close follow-up is recommended in cases of recurrent PUBA [7].

In the current case, we recommend the use of laser in treating multifocal lesions of PUBA. The use of laser is safe, and it maintains the capacity of the bladder without damage.

## Data Availability

All data generated or analyzed during this study are included within this article.
